# Clinical Arterial Peripheral Vascular Pathology Does Not Impact Short- or Long-Term Survival after Transcatheter Aortic Valve Replacement 

**DOI:** 10.1155/2018/2707421

**Published:** 2018-07-17

**Authors:** Brent Klinkhammer

**Affiliations:** University of North Dakota School of Medicine and Health Sciences, Grand Forks, ND 58202, USA

## Abstract

**Introduction:**

The dramatic changes in vascular hemodynamics after transcatheter aortic valve replacement (TAVR) are well noted. However, little postprocedural data exists on the outcomes in patients with clinical arterial peripheral vascular pathology [abdominal aortic aneurysm (AAA), carotid artery stenosis (CAS), and peripheral artery disease (PAD)] undergoing TAVR for severe aortic stenosis.

**Setting:**

A single center healthcare system.

**Methodology:**

A retrospective chart review case-control study of 342 consecutive patients who underwent a TAVR for severe aortic stenosis at Sanford Health in Fargo; ND was performed to determine if preprocedural comorbid AAA, CAS, or PAD was associated with worse outcomes after TAVR.

**Results:**

Patients with preprocedural comorbid AAA, CAS, or PAD had no significant difference overall survival at 1 month (94% versus 95% p =.812), 6 months (88% versus 89% p = .847), 1 year (74% versus 83%, p =.130), or 2 years (58% versus 63%, p =.611) after TAVR. Patients with clinical arterial peripheral vascular pathology also had no significant difference in preprocedural outcomes.

**Conclusion:**

This study gives evidence to suggest that patients with a comorbid clinical peripheral arterial pathology at the time of TAVR do not have a statistically significant increase in mortality out to 2 years after TAVR and no increase in procedural complications. These results affirm the safety and feasibility of TAVR in patients with AAA, CAS, and/or PAD.

## 1. Introduction

Previous studies have noted that minimally invasive interventional therapies have prominent and potentially clinical relevant effects on postoperative vascular hemodynamics [1, 2]. These effects are particularly prominent after transcatheter aortic valve replacement (TAVR). In a study by Yotti et al., it was demonstrated that the low systolic and pulse arterial pressure environment before TAVR was intensely and abruptly altered, leading to a significant increase in arterial pressures and vascular impedance after TAVR. This study also found a significant increase in systemic vascular resistance after TAVR, suggestive of an increase in arterial stiffness [1]. These post-TAVR hemodynamic changes have also been noted clinically in a study by Perlman et al. which found that 51% of patients who underwent a TAVR procedure either developed or had an interval worsening of their hypertension requiring additional pharmacotherapy postoperatively [3]. Interestingly, this study also found that post-TAVR exacerbation or development new hypertension after TAVR was associated with a decrease in in-hospital, 30 day, and 12-month serious adverse events [3].

However, Perlman et al. study leaves many unanswered questions about the long-term effects of the post-TAVR hypertension and changes in vascular hemodynamics. There remains a particular concern about “late” cardiovascular effects given these hemodynamic changes, their long-term vascular effects, and the effect on other comorbidities. For example, recent data has suggested that up to 30 percent of elderly patients suffer from cerebral “microbleeds” after TAVR [4]. In a similar way, the long-term effects of these vascular changes could be potentially detrimental in patients with preexisting chronic arterial disease.

Abdominal aortic aneurysm (AAA), carotid artery stenosis (CAS), and peripheral artery disease (PAD) are three common vascular disease which often coexists with aortic stenosis. The deleterious effects of hypertension on these disease states has been well noted [5–7]. However, the impact of aortic valve replacement on these disease is not well known. A previous study by Martinez-Selles, et al. found that PAD is associated with a lower survival rate after surgical aortic valve replacement [8]. Although 6-Minute Walk Test distance has been weakly associated with poor outcomes after TAVR AAA, CAS, and PAD have not been specifically associated with suboptimal outcomes after TAVR, but this has not been well-studied specifically [9, 10]. This study was designed to further define how the vascular effects of TAVR affect outcomes in patient with preprocedural clinically significant peripheral arterial pathology and if post-TAVR outcomes are affected by these comorbidities.

## 2. Methods

A hospital-based, single institution case-control study was conducted using data from one large Upper Midwestern integrated health system. We performed a retrospective chart review of 342 consecutive patients who underwent a transcatheter aortic valve replacement (TAVR) at Sanford Health in Fargo, ND, from 8/10/2012 to 11/15/2016 for severe aortic stenosis, defined as an aortic valve area less than 1 cm^2^. The last date of data acquisition was 1/4/2017. The entire cohort was divided into two groups where the patients preexisting AAA (> 3 cm), CAS [unilateral or bilateral stenosis >50% or carotid endarterectomy (CEA)], and/or symptomatic PAD were placed in one “vascular pathology” cohort, while all other patients were designated as controls. Primary outcomes were overall survival at 1 month, 6 months, 1 year, and 2 years after TAVR. Secondary outcomes were procedural complications, post-TAVR permanent pacemaker implantation, major adverse cardiovascular, and cerebrovascular events (MACCE) defined as death from any cause, myocardial infarction, rehospitalization, or stroke, cardiovascular mortality, myocardial infarction, stroke/TIA, heart failure exacerbation, or rehospitalization for any reason in defined time periods. Pre- and postprocedural echocardiographic data were also compared. The clinical outcomes were assessed in accordance with the standardized endpoint definitions for TAVR of the Valve Academic Research Consortium-2 [11]. Heart failure exacerbation was defined as a gradual or rapid change in heart failure signs and symptoms resulting in a need for a change in therapy or hospitalization.

Informed consent was not required for inclusion in our retrospective study due to the nature of the study and the absence of any direct interventions. This study protocol received dual IRB approval from the University of North Dakota IRB and from the Sanford Health IRB. Fisher's exact test was performed to determine statistical significance of categorical data and t-test or Wilcoxon two-sample test were used to determine the statistical significance continuous variables. All p-values were two-sided, and p-values < 0.05 were considered significant.

## 3. Results

A total of 147 of the 342 patients met study criteria for inclusion in the “vascular pathology” group. Baseline characteristics for both groups are given in Table 1. Statistically significant differences were noted in sex, STS risk score, EuroSCORE, preprocedural CAD, dyslipidemia, and baseline serum creatinine. There was also slight difference in the utilization of antiplatelet therapies at baseline. There was a high amount of significant comorbidities in both groups including an 88% prevalence of hypertension in the entire cohort. Mean age of the entire cohort was 79.2 years of age. Procedural characteristics for both groups are given in Table 2. There was no statistical differences in the specific type of valve used; however there was small, but statistical significant increase in the utilization of the transapical approach in the cohort that met study criteria. Pre- and postprocedural echocardiographic data are given in Table 3. Differences in peak aortic velocity and peak and mean aortic gradient were noted which were not sustained at 1 year after TAVR. A small statistically significant difference in aortic valve area was noted at 1 year after TAVR. Finally, the primary and secondary outcomes data for this study are given in Table 4. Overall survival for the entire study cohort was 79.5% at 1 year and 60.5% at 2 years.

## 4. Discussion

This study gives evidence to suggest that patients with a comorbid clinical peripheral arterial pathology at the time of TAVR do not have a statistically significant increase in mortality out to 2 years after TAVR. To our knowledge, this study is the first of its kind and demonstrates that, even in patients with a clinical peripheral arterial pathology and a high burden of other comorbidities, the changes in vascular hemodynamics after TAVR do not seem to confer any additional risk of morality or morbidity above the normal risk of TAVR. It should be noted that there does appear to be a nonsignificant trend towards higher mortality in the immediate time period, which is likely explained by the increased burden and comorbidities at baseline and likely not related to the effects of TAVR exacerbating underlying AAA, CAS, or PAD. The vascular pathology cohort in this study was more likely to be male, have underlying CAD and dyslipidemia, and have a higher STS risk score and EuroSCORE, which likely accounts for the mortality trend and also seemingly explains the statistically significant increase in major adverse cardiovascular and cerebrovascular events, cardiovascular death, and myocardial infarction seen in the 6 months to 1 year after TAVR time period.

This study also found some statistically significant differences in baseline and 24 hours after TAVR peak aortic velocity and peak and mean aortic gradient. This is likely of no clinical significance in that these differences were not maintained out to 1 year after TAVR and have not shown to be of any prognostic valve in the postoperative time period in other studies [12–14]. The vascular pathology cohort also had a slightly, but statistically significant, increase in the utilization of the transapical approach. This is unsurprising given that routine preoperative assessment of entire aorta, major thoracic arterial, carotids, and iliofemoral vasculature to rule out significant vascular disease or malformations often makes the transfemoral approach technically challenging and unfeasible. In our study, the transapical approach was necessitated in patients with clinical arterial pathology more often due to the nonfeasibility of the more preferred transfemoral approach secondary to significant stenosis, tortuosity, or other vascular complications. Previous studies have suggested that nontransfemoral approach may confer an increased risk of short- and long-term overall mortality [15]. However, despite this significant difference, there was no difference in any primary or secondary outcome to suggest less than optimal results in patient with underlying vascular pathology.

This study also supports the procedural safety of all TAVR approaches in patient with significant vascular disease. This study did not find any differences in direct procedural outcomes and suggests that the transfemoral approach is achievable in the vast majority of patients with AAA, CAS, or PAD. Large bore delivery sheaths used during the procedure increase the risk vascular complications including bleeding (especially retroperitoneal), vascular rupture, dissection, stenosis, perforation, arteriovenous fistula, pseudoaneurysm, irreversible nerve injury, or compartment syndrome that can lead to mortality and morbidity and often prompts additional interventions. This study did not find any increased risk of vascular complications as defined by the VARC-2 standards and no specific vascular complication occurred frequently [11]. There was also no significant differences in acute kidney injury or very early (<30 days after discharge) outcomes between the two groups.

Despite these reassuring findings, the impact of change in vascular hemodynamics after TAVR on microvascular disease remains in question. Our study is limited to largely macrovascular outcomes in a cohort at risk for macrovascular events. There remains considerable concern about the small vessel cerebral, renal, and ocular disease particularly in patient with preexisting disease or those with a predisposing condition like diabetes mellitus. For instance, Schmidt et al. found that female patients with diabetes mellitus have increased risk of embolic debris after implantation [16]. In another study, Khawaja et al. found that a history of diabetes mellitus, PAD, and chronic kidney disease were risk factors for post-TAVI acute kidney injury.[17]

This study does have some limitations including its retrospective design, single center experience, and inequalities in the length of post-TAVR follow-up. Like all retrospective analyses, the potential for confounding factors which were not identified and addressed in the study's baseline patient characteristics does exist. This study was designed to capture as many pertinent baseline characteristics as possible to effectively isolate the independent variable as much as possible. Patients in both groups were reasonably well matched overall. Unsurprisingly, however, there was an overall higher disease burden found in the vascular pathology group.

## 5. Conclusion

In this study, no association between the clinical arterial peripheral vascular pathology (AAA, CAS, and/or PAD) and a decrease overall survival was found. Additionally, the presence of arterial pathology at the time of TAVR did not confer an increased risk of procedural or early postprocedural complications. This study gives reassurance of the safety of TAVR in patient with significant peripheral vascular disease, although this conclusion is limited to macrovascular outcomes.

## Figures and Tables

**Table 1:** 

	Vascular Pathology (n=147)	Control (n=195)	P-value
**Age**	80.6 (7.55)	78.1 (9.76)	.011

**Male sex**	67 (99)	47 (92)	<.001

**BMI**	29.7 (6.04)	31.0 (6.34)	.344

**Caucasian race **	99 (145)	99 (194)	1.000

**EuroSCORE (**%**)**	10.91 (6.90)	6.84 (5.29)	<.001

**STS Risk Score (**%**)**	8.35 (4.67)	5.48 (3.03)	<.001

**Preprocedural HTN**	88 (129)	88 (172)	1.000

**Preprocedural CAD**	87 (128)	63 (123)	<.001

**Baseline Ejection Fraction <40**%	17 (25)	12 (24)	.275

**Preprocedural NYHA**	45 (66)	44 (85)	.827
**Class III OR IV Symptoms **			

**Preprocedural DM**	36 (53)	35 (68)	.820

**Prior Stroke/TIA**	16 (24)	8 (15)	.016

**Preprocedural Atrial Fibrillation **	35 (52)	27 (52)	.097

**Preprocedural Serum Creatinine (mg/dL)**	1.46 (1.14)	1.11 (0.46)	<.001

**Preprocedural eGFR < 60 mL/min**	55 (81)	43 (83)	.022

**Preprocedural Dyslipidemia**	100 (147)	83 (162)	<.001

**Preprocedural AAA**	26 (38)	-	-

**Preprocedural Carotid Artery **	63 (93)	-	-
**Stenosis >50**%** or Prior CEA**			

**Preprocedural Symptomatic PAD**	64 (94)	-	-

**Prior CABG**	35 (52)	23 (44)	.011

**Prior PCI**	44 (65)	31 (60)	.013

**Prior Permanent Pacemaker**	13 (19)	11 (22)	.737

**Prior Aortic Valvuloplasty**	19 (28)	16 (31)	.472

**Cardiovascular Pharmacology **			

** ACE inhibitor **	34 (50)	31 (61)	.641

** Angiotensin II receptor blocker **	14 (20)	21 (40)	.114

** Beta blocker (**%**)**	78 (114)	71 (138)	.174

** Calcium channel blocker **	26 (38)	30 (59)	.398

** Thiazide diuretic **	19 (28)	22 (43)	.590

** Loop diuretic **	52 (77)	47 (92)	.382

** Spironolactone **	3 (4)	4 (8)	.565

** Statin **	77 (113)	66 (128)	.031

** Aspirin**	84 (123)	70 (136)	.003

** Dual Antiplatelet Therapy**	36 (53)	22 (42)	.003

** Any anticoagulant **	28 (41)	23 (45)	.317

Values are mean (standard deviation) or % (n).

**Table 2:** 

	Vascular Pathology (n=147)	Control (n=195)	P-value
**Approach **			

** Transfemoral**	76 (111)	86 (167)	.025

** Transapical**	20 (29)	11 (21)	.030

** Transaortic**	1 (2)	3 (5)	.703

** Trans-subclavian**	3 (4)	1 (2)	.408

** Transcaval**	1 (1)	0 (0)	.430

**Mean LOS after TAVR (days)**	5.3 (9.4)	3.6 (3.2)	.077

**Valve type**			

** First generation Sapien**	23 (34)	30 (58)	.178

** Sapien XT**	17 (25)	13 (26)	.367

** Sapien S3**	32 (47)	34 (67)	.728

** First Generation CoreValve**	24 (35)	20 (39)	.427

** CoreValve Evolute**	4 (6)	3 (5)	.540

Values are mean (standard deviation) or n (%).

**Table 3:** 

	Vascular Pathology (n=147)	Control (n=195)	P-value
**Preprocedural aortic valve area (VTI) (cm** ^**2**^ **)**	0.88 (0.41)	0.87 (0.31)	.852

**Preprocedural peak aortic velocity (cm/s)**	401.8 (72.2)	424.8 (59.2)	.002

**Preprocedural peak aortic gradient (mmHg)**	67.2 (22.1)	73.8 (19.1)	.004

**Preprocedural mean aortic gradient (mmHg)**	42.7 (13.8)	46.3 (12.0)	.010

**Preprocedural ejection fraction (**%**)**	56.0 (13.6)	58.5 (12.0)	.068

**Pre-procedural stroke volume (mL)**	83.4 (18.6)	87.7 (21.9)	.094

**Preprocedural interventricular septum thickness (mm)**	12.5 (2.5)	12.5 (2.4)	.959

**Preprocedural moderate aortic regurgitation (**%**)**	20 (30)	19 (36)	.681

**Preprocedural severe aortic regurgitation (**%**)**	3 (5)	5 (9)	.784

**Preprocedural moderate mitral regurgitation (**%**)**	24 (36)	21 (41)	.514

**Preprocedural severe mitral regurgitation (**%**)**	3 (4)	4 (8)	.564

**24 hour post-TAVR aortic valve area (VTI) (cm** ^**2**^ **)**	2.26 (0.59)	2.15 (0.69)	.115

**24 hour post-TAVR peak aortic velocity (cm/s)**	212.6 (49.5)	228.5 (59.1)	.009

**24 hour post-TAVR peak aortic gradient (mmHg)**	19.0 (9.1)	22.2 (12.4)	.008

**24 hour post-TAVR mean aortic gradient (mmHg)**	10.9 (5.5)	13.4 (7.6)	.001

**24 hour post-TAVR ejection fraction (**%**)**	60.8 (13.2)	61.6 (12.4)	.583

**24 hour post-TAVR stroke volume (mL)**	91.9 (25.0)	96.0 (29.5)	.204

**24 hour post-TAVR moderate aortic regurgitation (**%**)**	5 (7)	6 (11)	.810

**24 hour post-TAVR moderate mitral regurgitation (**%**)**	11 (16)	9 (18)	.590

**24 hour post-TAVR severe mitral regurgitation (**%**)**	3 (4)	2 (3)	.467

**1 year post-TAVR aortic valve area (VTI) (cm** ^**2**^ **)**	2.13 (0.63)	1.91 (0.57)	.039

**1 year post-TAVR peak aortic velocity (cm/s)**	217.1 (47.5)	223.0 (51.2)	.489

**1 year post-TAVR peak aortic gradient (mmHg)**	20.3 (10.3)	20.9 (10.0)	.733

**1 year post-TAVR mean aortic gradient (mmHg)**	11.4 (5.9)	12.0 (5.9)	.579

**1 year post-TAVR ejection fraction (**%**)**	57.5 (13.6)	58.5 (12.6)	.661

**1 year post-TAVR stroke volume (mL)**	96.0 (26.3)	91.8 (29.7)	.411

**1 year post-TAVR moderate aortic regurgitation (**%**)**	9 (5)	16 (14)	.314

**1 year post-TAVR moderate mitral regurgitation (**%**)**	17 (10)	9 (8)	.197

**1 year post-TAVR severe mitral regurgitation (**%**)**	9 (5)	3 (3)	.264

Values are mean (standard deviation) or %.

**Table 4:** 

	Vascular Pathology	Control	P-value
%** Survival > 1 month**	94 (138/147)	95 (186/195)	.812

%** Survival > 6 month**	88 (102/116)	89 (142/159)	.847

%** Survival > 1 year**	74 (69/93)	83 (110/132)	.130

%** Survival > 2 year**	58 (37/64)	63 (52/83)	.611

**Periprocedural Major Vascular**	10 (14)	8 (15)	.561

**Periprocedural Minor Vascular**	10 (15)	8 (15)	.443

**Post-TAVR PPM implantation **	8 (12)	8 (16)	1.000

**Periprocedural Increase in Serum Creatinine >1.5x baseline**	8 (12)	4 (7)	.093

**In Hospital CV Mortality**	6 (9)	6 (11)	.821

**In Hospital MI**	0 (0)	1 (2)	.509

**In Hospital Stroke/TIA**	2 (3)	3 (6)	.738

**In Hospital HF exacerbation**	24 (35)	19 (38)	.352

**Discharge to 30 days MACCE**	18 (24)	18 (33)	1.000

**Discharge to 30 days CV Mortality **	0 (0)	1 (1)	1.000

**Discharge to 30 days Myocardial Infraction **	1 (1)	1 (2)	1.000

**Discharge to 30 days Stroke/TIA**	1 (1)	1 (2)	1.000

**Discharge to 30 days HF exacerbation**	17 (23)	15 (28)	.758

**Discharge to 30 days Rehospitalization For Any Reason**	17 (23)	18 (33)	.882

**30 days- 6 months MACCE**	31 (32)	25 (37)	.389

**30 days- 6 months CV Mortality**	2 (2)	2 (3)	1.000

**30 days- 6 months Myocardial Infraction**	2 (2)	1 (2)	1.000

**30 days- 6 months Stroke/TIA**	3 (3)	2 (3)	.694

**30 days- 6 months HF exacerbation**	17 (18)	14 (20)	.477

**30 days- 6 months Rehospitalization For Any Reason**	28 (29)	22 (33)	.373

**6 months-1 year MACCE**	40 (30)	24 (26)	.033

**6 months-1 year CV Mortality**	5 (4)	0 (0)	.028

**6 months-1 year Myocardial Infraction**	5 (4)	0 (0)	.028

**6 months-1 year Stroke/TIA**	4 (3)	0 (0)	.069

**6 months-1 year HF exacerbation**	17 (13)	21 (22)	.703

**6 months 1 year Rehospitalization For Any Reason**	33 (25)	26 (24)	.240

Values are % (n).

MACCE = major adverse cardiovascular and cerebrovascular events, defined as death from any cause, myocardial infarction, rehospitalization, and stroke

## Data Availability

The data used to support the findings of this study are available from the corresponding author upon request.
